# Modeling the potential impact of storm surge and sea level rise on coastal archaeological heritage: A case study from Georgia

**DOI:** 10.1371/journal.pone.0297178

**Published:** 2024-02-28

**Authors:** Matthew D. Howland, Victor D. Thompson

**Affiliations:** 1 Department of Anthropology, Wichita State University, Wichita, KS, United States of America; 2 Department of Anthropology, University of Georgia, Athens, GA, United States of America; University of Strathclyde, UNITED KINGDOM

## Abstract

Climate change poses great risks to archaeological heritage, especially in coastal regions. Preparing to mitigate these challenges requires detailed and accurate assessments of how coastal heritage sites will be impacted by sea level rise (SLR) and storm surge, driven by increasingly severe storms in a warmer climate. However, inconsistency between modeled impacts of coastal erosion on archaeological sites and observed effects has thus far hindered our ability to accurately assess the vulnerability of sites. Modeling of coastal impacts has largely focused on medium-to-long term SLR, while observations of damage to sites have almost exclusively focused on the results of individual storm events. There is therefore a great need for desk-based modeling of the potential impacts of individual storm events to equip cultural heritage managers with the information they need to plan for and mitigate the impacts of storm surge in various future sea level scenarios. Here, we apply the Sea, Lake, and Overland Surges from Hurricanes (SLOSH) model to estimate the risks that storm surge events pose to archaeological sites along the coast of the US State of Georgia in four different SLR scenarios. Our results, shared with cultural heritage managers in the Georgia Historic Preservation Division to facilitate prioritization, documentation, and mitigation efforts, demonstrate that over 4200 archaeological sites in Georgia alone are at risk of inundation and erosion from hurricanes, more than ten times the number of sites that were previously estimated to be at risk by 2100 accounting for SLR alone. We hope that this work encourages necessary action toward conserving coastal physical cultural heritage in Georgia and beyond.

## Introduction

### Climate change, sea level rise, and coastal archaeology

Anthropogenic climate change is one of the most pressing challenges of our time. At particular risk are coastal environments due to the inevitability of sea level rise (SLR). Future scenarios depend on the extent to which emissions are curbed, with 0.6 m of SLR by 2100 likely and as much as 1.1–2.1 m possible in a business-as-usual-scenario [1]. Climate change also will likely drive an increase in the severity of tropical cyclones, ultimately resulting in stronger storm surges [2–5]. The proportion of tropical cyclones that reach Category 4 and 5 on the Saffir-Simpson scale is projected to increase as a result of these changes [6]. These shifts will inevitably have a drastic effect on coastal communities, forcing large-scale adaptation and migration [7,8].

In addition to impacts on living populations, SLR will also threaten coastal historic and archaeological sites that represent the physical cultural heritage of our communities [9]. Sites in low-lying coastal regions are at increasing risk of erosion, inundation, and submersion as sea levels continue to increase in the near future. This inevitable process requires attention from cultural heritage professionals, who are uniquely qualified to address threats to historic and archaeological sites [10]. Mitigation of the impact of SLR on heritage sites requires a multi-stage, collaborative process, including vulnerability assessments, prioritization, documentation, and conservation, which should be urgently implemented based on increasing risks to coastal sites [11]. This work must involve close consultation and collaboration with stakeholders, especially including Indigenous and descendant community groups [12,13]. Moreover, vulnerability assessments, when conducted, should be made available to cultural heritage managers and decision makers in order to facilitate mitigation efforts. These assessments are a key first step in providing cultural heritage managers with the information needed to plan for future risks to physical cultural heritage.

Many studies have examined the risks posed by sea-level rise to coastal heritage sites. These studies typically attempt to model future impacts, describe ongoing or past impacts, or both. Desk-based modeling efforts have ranged in complexity and scale, from applications of global and regional SLR projections, including simple “bathtub-style” models [14–17] or more sophisticated models that account for local shoreline dynamics [10,18–20]. The latter category includes use of the Sea Level Affecting Marshes Model (SLAMM), which allows for nuanced desk-based modeling of how ecological niches and land cover will change in response to rising sea levels [21,22]. Largely, these SLR modeling approaches, regardless of complexity, aim to understand the gradually-occurring impacts caused by SLR on coastal environments, including submersion, inundation, and related erosion. SLR is an attractive subject for desk-based modeling as it is a predictable process that can be modeled (though without exact precision) according to known factors [23]. Complementing the many published desk-based models of SLR are a number of corresponding studies examining the actual impacts of coastal erosion processes and tidal variation on coastal heritage sites [24–28]. These studies provide valuable ground truthing of how medium to long term processes of SLR will likely impact archaeological heritage. Decision makers are therefore well-equipped with information to understand the risks posed by accelerating SLR over the next century.

**Fig 1:**
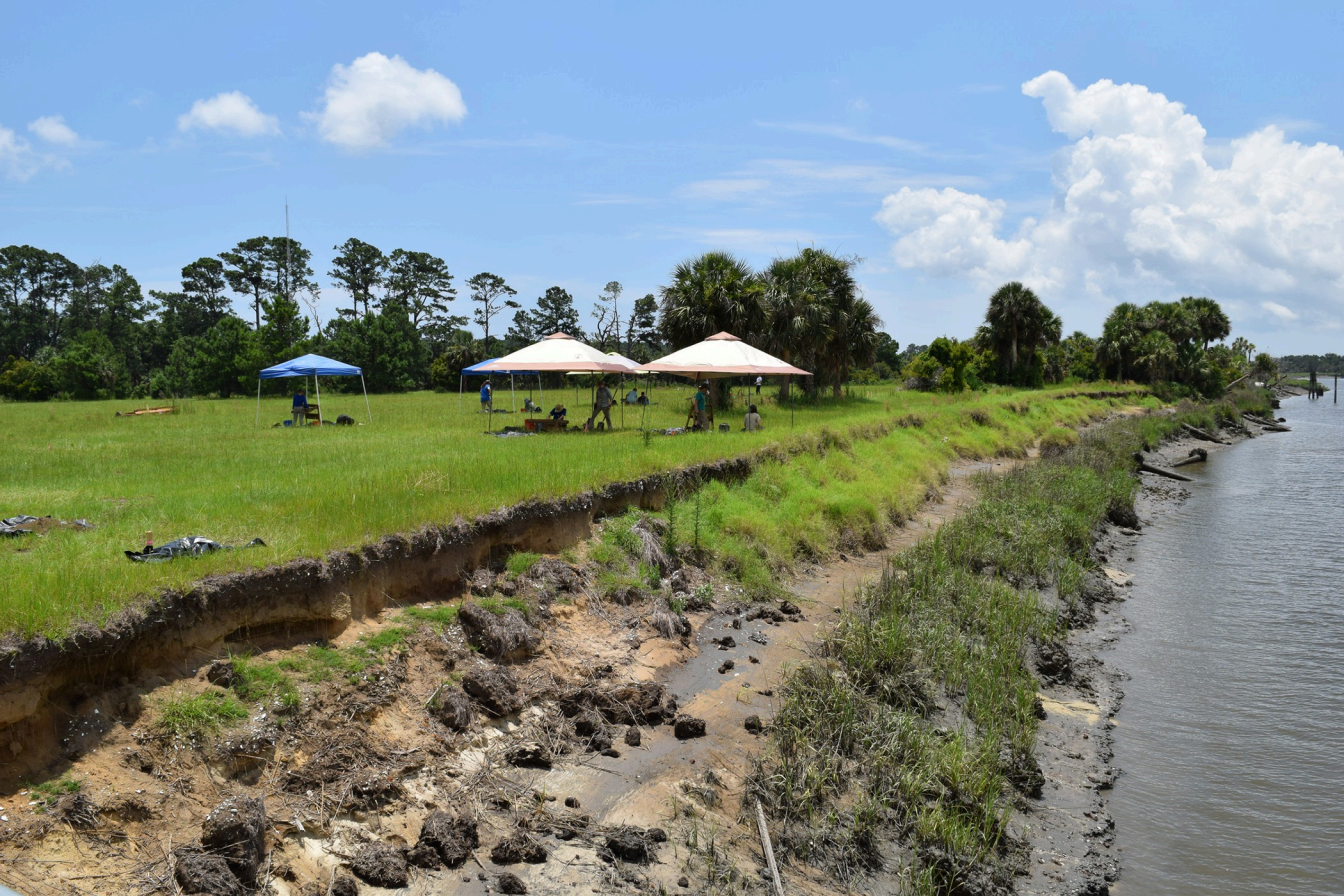
Erosion at the South End site on Ossabaw Island, Georgia, caused mainly by storm surge from Hurricane Michael.

However, most studies examining ongoing or past damage to archaeological sites tend to identify erosional impacts caused by specific storm events rather than longer term processes caused by SLR [12,16,29–36] (Fig 1). The ubiquity of ground-based studies identifying damage from specific storms implies that major storms have an outsized influence in posing risks to coastal archaeological sites. This suggests a need for desk-based modeling and spatial quantification of potential storm surge scenarios. Yet, as pointed out by Rivera-Collazo [34], very little work has been done to quantify this threat—only a limited number of studies have applied desk-based models to predict the impacts of future storm surge on heritage sites [37–40].

There is therefore an inconsistency between the desk-based modeling of coastal heritage risk—nearly always focused on gradual processes of SLR and coastal erosion/accretion processes—and field-based observation of eroded and damaged sites, often conducted in response to individual storm events. This disparity presents a challenge for cultural heritage managers planning mitigation efforts. The ubiquity of literature reporting on the severe impacts of individual storm events suggests a need for cultural heritage managers to have access to vulnerability assessments for emergency events in order to be able to develop mitigation plans well in advance of potential catastrophic events. Desk-based modeling of various disaster scenarios, when provided to decision makers, can be used as the basis for both heritage risk mitigation and disaster relief efforts focused on physical cultural heritage.

### Modeling storm surge

Modeling storm surge is an extremely complex process [41]. The physics of storm surge depend on storm size [42], speed [43], wind fields [44], coastal topobathymetry [45], tides [46], and land cover [47], among other factors. This complexity means that forecasting specific storm surge events with any degree of reliability is impossibly challenging, given that small variations in storm track, intensity, or tidal conditions could greatly impact the intensity of flooding and inundation. Moreover, the sophistication of storm surge models means that cultural heritage professionals typically will not possess the requisite knowledge and technical ability to develop custom storm surge prediction models. Heritage practitioners must instead rely on models developed by other researchers or agencies [48–50].

One such model is the Sea, Lake, and Overland Surges from Hurricanes (SLOSH) model, developed by the National Weather Service (NWS) as a tool to estimate storm surge heights from various hurricane scenarios within +/- 20% of peak storm surge [51]. Outputs from this model are available to users through the SLOSH Display Program, in which storm surge projections generated by the SLOSH model based on input storm conditions are made available to the public. The display program provides a customizable interface, allowing users to select a storm basin along with characteristics such as category of storm, speed, direction, and tidal conditions. The program also provides multiple modeling approaches, including displays based on the specific physics of a potential storm or a composite approach intended to model general storm surge vulnerability. Applying this latter approach produces either Maximum Envelopes of Water (MEOWs; i.e., the highest storm surge likely in a particular grid square given certain storm conditions) or Maximum of MEOWs (MOMs; i.e., a composite of the highest storm surge likely in a particular grid square across many storms in a given category on the Saffir-Simpson scale). SLOSH-MEOWs therefore represent a worst-case scenario for a particular storm, whereas SLOSH-MOMs represent an overall worst-case scenario for the entire basin. Data from either model can be exported in various formats from the SLOSH Display Program, including as shapefiles providing the height of storm surge above a vertical datum. This surge height can be compared to local elevation data to determine areas of inundation. The SLOSH display program also provides multiple visualization options, including animations of modeled storm surges. Alternatively, SLOSH projections are also available as raster data for storm categories through the NWS’ National Storm Surge Risk Maps [52]. Raster data from the National Storm Surge Risk Maps is already corrected for local elevation, meaning that values are given in storm surge heights above ground elevation rather than above the arbitrary datum. Therefore, acquiring SLOSH-MOMs data directly from the National Storm Surge Risk Maps is more efficient for understanding present day storm surge risk, but is less versatile for developing more complex models.

The SLOSH model has been applied to a limited extent by cultural heritage professionals, primarily to model the extent to which coastal sites are at risk from storm surge in a context of climate change and SLR. In particular, this model has been used to understand potential impacts on historic cemeteries in Florida [37], archives in the US [53], and heritage sites in North Carolina [39] and the Delaware Bay [40]. These studies illustrate the utility of applying the SLOSH model for projecting potential impacts of storm surge on heritage sites, providing a basis for preparing for the type of specific impacts on coastal heritage sites from major storms that are often identified by archaeologists.

### Coastal heritage in Georgia

**Fig 2:**
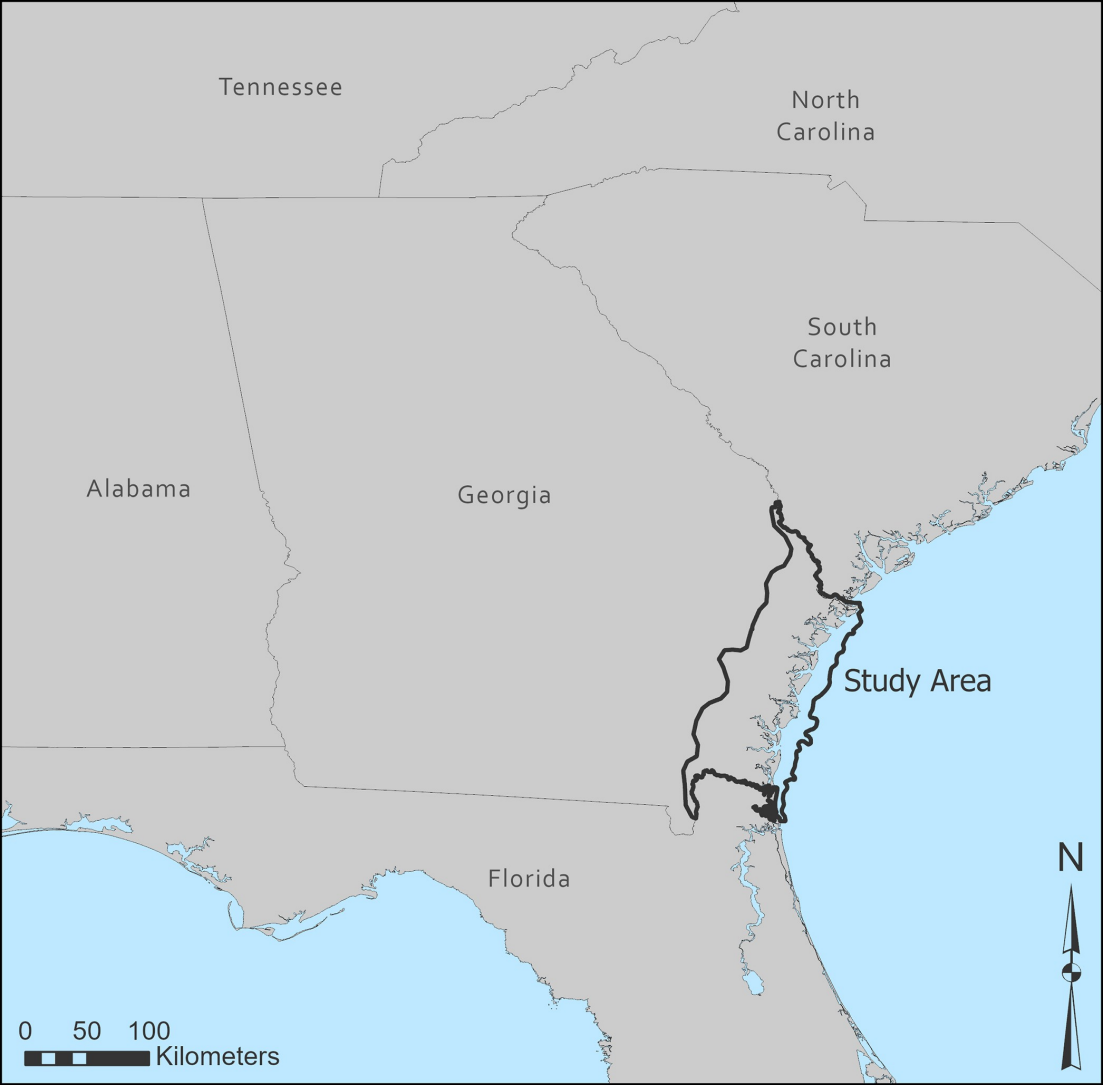
The study area, consisting of the Georgia coast and ca. 50 km inland.

The US State of Georgia provides an excellent case study for the projection of potential storm surge impacts on coastal heritage sites because of the state’s flat coastal plain and long history of occupation of coastal areas and exploitation of marine resources [54,55] (Fig 2). Georgia’s coastal geomorphology consists of at least six coastal “terraces” within 30 m of present-day mean sea level (MSL), reflecting ancient Pleistocene coastlines that are the result of changing sea levels over time. The deposits making up these terraces accumulated in barrier island and lagoon salt marsh contexts that are likely similar to the present environmental context [56–58]. Ecological niches along the Georgia coast are well-buffered for processes of gradual SLR due to the high tidal ranges and high sedimentation and accretion rates along the coast [59,60]. For example, salt marshes can accumulate sediment and migrate inland in rising sea level conditions [61]. The persistence of this ecosystem against sea level change may be one contributing factor to the resilience shown by Ancestral Muskogean people who inhabited sites on the Georgia coast and adapted to fairly drastic environmental and sea level fluctuations [55,62,63].

However, local sea level conditions and high tidal ranges also make Georgia more susceptible to SLR than other parts of the world. Georgia is likely to see higher SLR than global mean sea level rise [1], and high tidal ranges mean that even higher upland sites are likely to be inundated during ordinary tidal conditions. Furthermore, the particular adaptability of the coastal ecosystem in Georgia means that ongoing erosion and sedimentation processes pose serious risks to archaeological sites in the region. In addition to important Indigenous Native American cultural sites, the Georgia coast is host to a number of culturally significant Spanish missions, Euromerican plantations, and colonial forts. These sites tend to be located along the edges of tidal creeks, marshlands, and coastal sounds, making them particularly vulnerable to SLR. Robinson and colleagues [24,27] found that 11 of 21 sites in coastal Georgia studied were actively eroding, providing an example of the slow, steady, and sure heritage risk posed by SLR and often modeled at regional scale [14]. Recent analysis by Cochran and colleagues [22] using the SLAMM model to predict how SLR will impact coastal ecology also demonstrates that ca. 40% of archaeological sites along the Georgia coast are at risk of context loss related to marsh migration to upland zones.

In addition to impacts from SLR, sites on the Georgia coast are also at great risk of impacts from storm surge. The state’s wide, low coastal plain is predisposed to inundation and flooding from major storms, despite the attenuating impact of tidal marshes on storm surge [64–66]. Georgia has a relatively long recorded history of storms causing major flooding, with recording of storm surge heights dating back to 1804 [67]. One particularly severe storm occurred in October 1898, when a Category 4 hurricane made landfall on Cumberland Island and caused ca. 16+ feet of storm surge [68], inundating the second stories of buildings along coastal barrier islands [69]. Fortunately, no storm greater than a Category 2 Hurricane has made landfall on the Georgia coast since. However, given the increased likelihood of increasingly severe storms, preparing for the potential impacts of storm surge on heritage sites is imperative.

## Methods

### Sea level rise and storm surge modeling

Our approach models 24 storm surge scenarios, consisting of six storm surge scenarios corresponding to the five levels of the Saffir-Simpson scale (1–5) and a non-hurricane tropical storm, modeled for each of four SLR scenarios. The four potential sea level conditions modeled reflect present sea level and high (Global Mean SLR rise of 2.0 m), medium (1.0 m), and low (0.3 m) SLR scenarios for 2100 from the NOAA Sea Level Rise Technical Report [1]. These possibilities were adapted to the local conditions of the Georgia coast through use of the US Army Corps of Engineers Sea Level Tracker [70] based on projections for the NOAA tidal monitoring station at Fort Pulaski, GA, near Savannah, GA. This adjustment resulted in high SLR of 2.09 m, moderate SLR of 1.38 m, or low SLR of 0.5 m along the Georgia coast by 2100. Note that in each case, local SLR projections for Georgia exceed global mean SLR values, illustrating the particular susceptibility of the Georgia coast to SLR. These projections were subtracted from high-resolution coastal topobathymetric data from the U.S. Geological Survey’s Coastal National Elevation Database Applications Project [71] in order to provide a baseline from which to compare inundation depths produced by the SLOSH model.

Understanding future risks of storm surge requires not only projection of SLR, but also modeling of coastal inundation under various storm conditions. For this purpose, we apply SLOSH-MOMs for worst-case scenarios for each of the five storm categories in the Saffir-Simpson scale. This conservative approach (i.e., using SLOSH-MOMs rather than SLOSH-MEOWs) aims to ensure that the model does not underestimate the impact of a particular storm on cultural heritage sites, at the expense of providing estimates that do not correspond to any possible individual storm [52]. Though raster data from the NWS’ National Storm Surge Risk Maps is easily available and applied, we leverage data from the SLOSH Display Program in order to more easily combine storm surge modeling with the SLR projections discussed above. We have converted the shapefile output of the SLOSH Display program to raster and converted units from feet to meters, before accounting for the possibility of the SLR scenarios described above by subtracting surge heights calculated by SLOSH from the adjusted coastal topobathymetric data and reclassifying the resulting dataset to determine which areas along the Georgia coast would be inundated under each SLR and storm surge scenario. These data conversions were performed using ArcPy in ArcGIS Pro, and code for the analyses conducted is available at 10.5281/zenodo.10079835.

These methods faithfully reconstruct the vast majority of inundation due to SLR and storm surge along the coast. However, since SLOSH Display Program output shapefiles only contain storm surge height data for inundated areas (based on present sea level conditions), our approach is not able to account for areas along the coast that would remain above the waterline (i.e., not flooded) in even the most severe storms at present sea level but that are likely to be inundated by severe storms under conditions of rising sea level. To account for this systematic underestimation of inundation, we assume that archaeological sites with elevations below the combined SLR projection and maximum storm surge height (by storm category) across the entire study area are potentially at risk, despite uncertainty regarding how local geomorphology would affect storm surge dynamics in these areas. Our code, provided at 10.5281/zenodo.10079835, contains a correction for this underestimate. On a related note, the approximation of inundated areas under various conditions in this study does not account for the extent to which changes in coastal geomorphology (i.e., erosion and accretion) caused by SLR would impact storm surge conditions along the coast in future SLR scenarios. This means that, like other studies using the SLOSH model and SLR projections (e.g., [40]), our methods potentially underestimate the extent to which storm surges in future SLR scenarios will inundate inland areas. Undoubtedly, changing topobathymetry due to SLR will have significant impacts on the localized effects of storm surges [45]. Still, the 24 modeled scenarios produced through this methodology ultimately provide comprehensive baseline projections of potential storm surge events under different sea level conditions occurring now and in 2100.

### Georgia’s natural, archaeological, and historic resources GIS

The SLR and storm surge model developed above allows for comparison with known cultural heritage sites as part of a vulnerability assessment. In the state of Georgia, the locations of recorded historic and archaeological sites are curated through Georgia’s Natural, Archaeological, and Historic Resources GIS (GNAHRGIS), a spatial database operated by the Georgia Archaeological Site File (GASF) at the Laboratory of Archaeology at the University of Georgia. GNAHRGIS contains spatial data for over each archaeological site recorded in the GASF, as well as information on historic sites identified by the National Register of Historic Places and the Georgia Historic Resources Survey Program across the state. Accessing cultural heritage site locations through this comprehensive database provides an excellent basis for understanding which archaeological and historic sites are vulnerable to impacts related to SLR [22]. Access to this database was provided by the GASF in order to conduct this research. Here, use of GNAHRGIS allowed a storm surge-based site vulnerability assessment to be conducted by converting each of the 20 scenarios of storm surge inundation from raster to vector and then identifying intersections between inundated areas and archaeological and historical sites. This process allows for understanding how many of the 5,574 archaeological sites in our study area (Fig 2) along the Georgia coast would potentially be subject to inundation by storm surge in various storm surge and SLR scenarios.

This modeling was developed in collaboration with the Historic Preservation Division (HPD) of the Georgia Department of Community Affairs. The HPD functions as Georgia’s state historic preservation office (SHPO). This collaboration with a key state agency responsible for management and conservation of cultural heritage sites at the early stage of vulnerability assessment allows for results of this analysis to be more easily implemented into planning, prioritization, and mitigation efforts. In this case, collaboration with the Georgia HPD allows these storm surge and SLR projection models to be published on GNAHRGIS. These models are therefore available to stakeholders across the state with appropriate levels of permissions to access archaeological site data on GNAHRGIS, while coarser storm surge projection models (at 1000 m spatial resolution rather than 10 m) are freely available to the general public at https://usg.maps.arcgis.com/apps/instant/portfolio/index.html?appid=0f5ac94888124ed48dc7968703f48d4e. Users can identify the historic and archaeological sites that are potentially at risk in various storm surge and SLR scenarios by displaying multiple data layers in the GNAHRGIS user interface. Similarly, stakeholders with appropriate levels of permissions to access archaeological site data will be able to identify specific sites that are vulnerable based on this modeling. Collaboration with the Georgia HPD therefore increases access to vulnerability assessments both among the general public and decision makers in state government.

## Results and discussion

**Fig 3:**
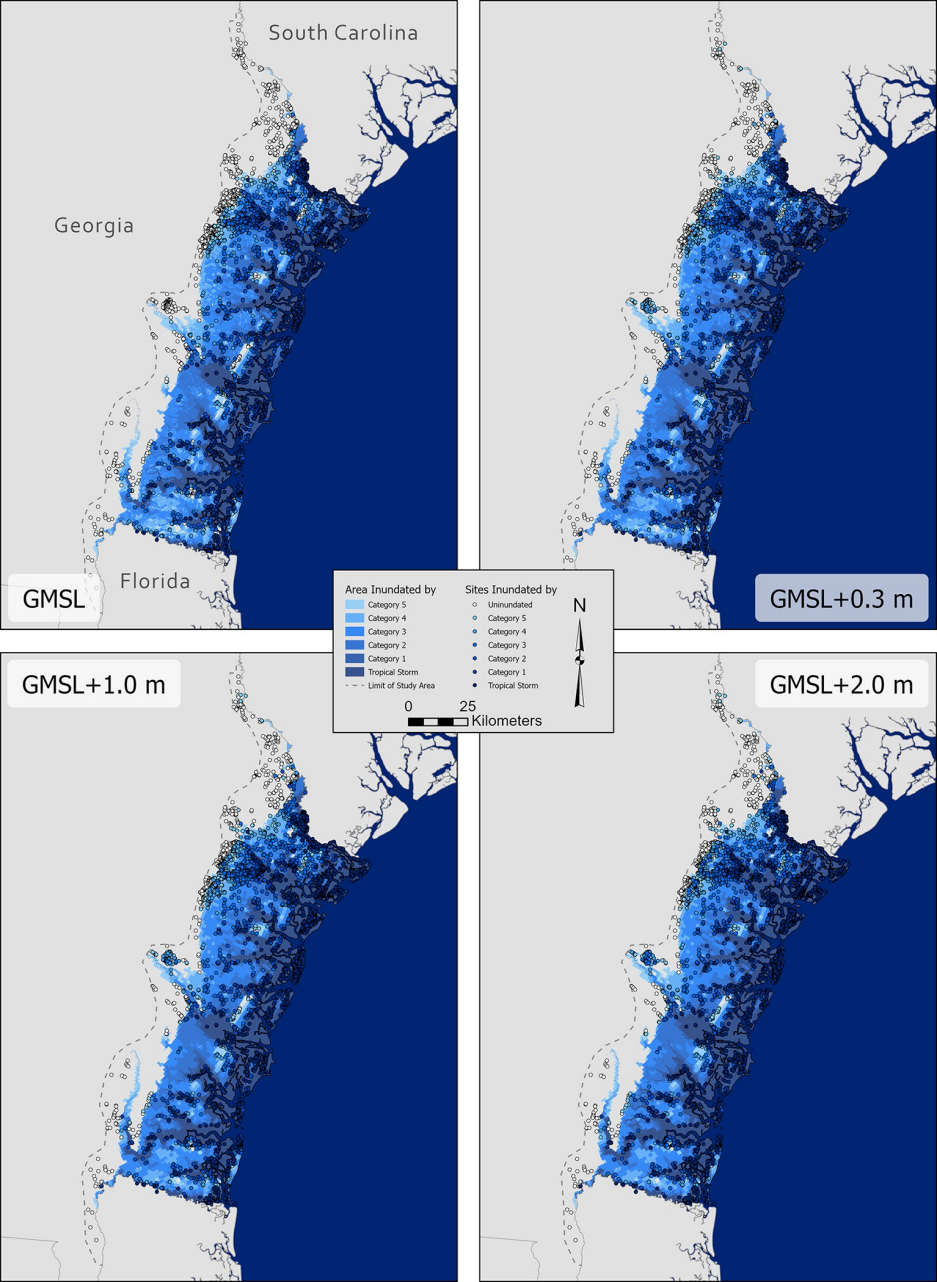
Worst-case scenarios for storm surge inundation according to six storm surge conditions (tropical storm and categories 1–5 on the Saffir-Simpson Scale) for four SLR scenarios (present sea level, + 0.3 m GMSL, +1.0 GMSL, and +2.0 GMSL) adjusted for the Georgia coast. Note that these projections may underestimate inundated areas, especially under increasingly severe SLR projections.

The results of this analysis illustrate the potentially severe impact that storm surge events could have on coastal heritage sites in the state of Georgia (Fig 3; Table 1). Due to the relatively flat and wide topography of the coastal plain of Georgia, much of the coastal region, including the city of Savannah, is at risk from surge events from hurricanes at or above Category 3 on the Saffir-Simpson scale. This includes most of Georgia’s barrier islands, the back barrier, tidal marshes, and ca. 15 km of mainland. The number of archaeological sites projected to be inundated by storm surge events demonstrates the comparatively devastating impact that these events can have when compared to the relatively slow process of SLR. A Category 5 hurricane at present sea level, for example, could inundate over 4200 archaeological sites on the Georgia coast—more than 75% of the sites in the study area—according to this SLOSH-MOM modeling. By comparison, a high SLR projection of 2m by 2100, reflecting what would essentially be a worst-case scenario, would only inundate 397 archaeological sites [14]—less than half of the sites potentially impacted by storm surge caused by a Tropical Storm at present sea level. These results illustrate that archaeological sites in coastal regions are at far greater risk of inundation under current conditions than previously believed based on models accounting for only SLR processes. Accounting for storm surge events, which could occur at any time, means that more than ten times as many archaeological sites are at risk of inundation and potentially erosion and accretion than even worst-case projections of site risk from SLR alone by 2100.

**Table 1:** Number of archaeological sites in Georgia potentially inundated according to different SLR and storm surge projections.

	Tropical Storm	Category 1 Hurricane	Category 2 Hurricane	Category 3 Hurricane	Category 4 Hurricane	Category 5 Hurricane
**Present Sea Level**	953	1540	2661	3561	4058	4290
**GMSL + 0.3 m**	1201	1791	2852	3705	4193	4658
**GMSL + 1.0 m**	1615	2082	3057	3866	4309	4791
**GMSL + 2.0 m**	1898	2250	3191	3967	4397	4892

Our analysis projects that the vast majority of archaeological sites within 40km of the Georgia coast are currently at risk of damage through inundation, erosion, or accretion from storm surge events that, though unpredictable, could occur at any time. Sites at risk include, for example, the several Late Archaic (5000–3000 BP) shell ring villages once inhabited by Ancestral Muskogean people, such as the Sapelo Shell Ring Complex, along the Georgia coast, that have been instrumental for understanding monumentality and feasting [72] and collective action [73,74] among foraging societies and, fittingly, resilience in response to climatic instability and rapid sea level change [55,63]. These coastal sites, representing important physical cultural heritage of the Muscogee Nation and other Federally recognized tribes, are at imminent risk from potential storm surge events—the Sapelo Shell Ring Complex, for example, would be inundated by storm surge from a Category 2 hurricane at present sea level, or as little as storm surge from a Category 1 hurricane in our projection for GMSL +1.0 m. Also at risk are sites associated with the early colonial history of Georgia, such as Fort King George, an early 18^th^ century CE English fort [75,76] that would be inundated by storm surge caused by a mere tropical storm at present day sea level, according to our model. Similarly, Santa Catalina de Guale, a 17^th^ century Spanish Franciscan mission that evidences the northern extent of Spanish colonization endeavors on the Georgia Coast and Indigenous resistance to these efforts [77,78], could be impacted by as little as a Category 2 hurricane in each SLR scenario, we project. These examples, along with the more than 4200 other archaeological sites that our analysis shows are at potentially imminent risk of inundation from storm surge events, represent a broad cross section of the archaeological past and Georgia’s history that is in danger of being damaged or lost.

As such, our analysis indicates that a paradigm of projecting risks to coastal archaeological sites based on modeling relatively long-term processes of increased MSL without factoring in storm surge impacts systematically underestimates the risk faced by archaeological sites under sea level conditions in the present and near future. This observation is borne out through ground-based study of the impacts of storm events on coastal heritage sites [34]. On a similar note, our work suggests that the majority of risk posed to archaeological sites is driven by the unknown possibility of storm surge events rather than the certainty of gradual SLR, even under relatively severe projected SLR scenarios (Table 1). Importantly, this vulnerability assessment does not account for site-specific factors that impact or damage heritage sites, which often requires ground-based evaluation. Indeed, a site being inundated or submerged does not necessarily mean that it has been damaged [79]. This caveat applies to desk-based modeling of both SLR and storm surge. Moreover, this analysis only accounts for known sites—likely many unknown sites are at risk of inundation and potential damage in each of the scenarios analyzed here.

Focusing specifically on the potential impacts of storm surge on the Georgia coast, according to these projections, inundation risks are limited to areas below the Talbot Formation, one of the six Pleistocene paleoshorelines along the Georgia coast. The rise in elevation associated with this formation limits the extent to which potential storm surge events can affect archaeological sites and areas further inland without drastic SLR scenarios greater than projected by 2100. Ridges corresponding with Pleistocene shorelines at lower elevations (e.g., the Pamlico, Princess Anne, and Silver Bluff Ancient Coastal Deposits (ACDs) similarly impact the susceptibility of areas along the coast to less severe storm surge events. The spatial limitation offered by the Talbot ACD to the potential impacts of any foreseeable SLR and storm surge scenario provides a clear geographic area for cultural heritage managers to focus documentation and mitigation efforts.

Despite the limited impact of SLR on projections for severe storms, rising sea levels are likely to have a comparatively large influence on the extent to which archaeological sites are impacted by smaller storms. At particular risk are barrier islands, backbarrier and tidal marshes, tidal channels, and river deltas. These contexts generally represent extremely low-lying topography, common along the Georgia coast and already at risk from documented ongoing coastal erosion processes (cf. [27]). Generally, backbarrier and tidal marshes are likely to be inundated through any projected storm surge, an unsurprising result given these wetlands already experience regular inundation. Barrier islands, therefore, experience the greatest potential negative impact from future SLR scenarios. At present sea level, these islands are at risk from inundation, but largely only from more severe storms. In future SLR scenarios, even surges from tropical storms can potentially inundate most of the dry land on Georgia’s 14 major barrier islands, putting the hundreds of archaeological sites on these islands at risk. SLR is also likely to have a great impact on the extent to which floodplains of rivers and streams along the Georgia coast are subject to inundation from even minor storms (Fig 3D).

Analysis of these storm surge and SLR projections allows for an informed assessment of the extent to which archaeological sites along the Georgia coast are vulnerable to individual future storms. This information is essential for cultural heritage managers to plan and implement prioritization, documentation, and conservation efforts for various potential future storm categories. Planning for storm surges from tropical storms or more severe hurricanes making landfall elsewhere is of greatest immediacy. These storm events are likely to occur in the near future and are well-attested in the historical record of the Georgia coast [67,69], as discussed above. Storm surge from a tropical storm, relatively likely to occur in the coming decades, would currently threaten 953 archaeological sites on the Georgia coast and as many as 1898 sites by 2100, according to our projections. These predictable risks suggest an urgent need to document this at-risk heritage. In particular, efforts to comprehensively survey both known and unknown sites that are at risk from damage from erosion and potentially permanent submersion are needed. Publication of projections from this analysis on the GNAHRGIS platform also provides decision makers in local and state government with the information needed to prioritize sites for potential salvage excavation and/or mitigation efforts to protect key sites against damage from these predictable effects of lesser storms. Such prioritization and planning should be conducted in partnership with local communities and stakeholders [12,13,34].

Vulnerability assessment also includes analyzing the risk posed by more severe storms, which are less predictable but are a looming threat over longer timescales. Since climate change is driving an increase in sea levels and the severity of hurricanes and storm surges [1–5], more severe impacts on archaeological sites are increasingly likely to occur and must be accounted for by decision makers. Between 953 and 4892 archaeological sites are potentially at risk in future hurricane-based storm surge events, potentially catastrophic for both living communities along the Georgia coast and the archaeological record. These results suggest that accounting for the risk posed by individual storm events to coastal archaeological sites is a critical component of quantifying the risk of climate change on these physical cultural heritage sites, despite a prior tendency to focus on gradual processes of SLR alone. Unfortunately, mitigation of the most severe potential storm surge events is an enormous challenge for society at large. The scale of these events likely exceeds the ability of cultural heritage professionals to protect archaeological sites. Still, advance preparation for these potential disaster events is necessary for the development of emergency action plans to document damaged or destroyed heritage sites. SLR and future storm surge events pose major risks to our society and physical cultural heritage and desk-based vulnerability assessments are a key first step toward developing action plans for mitigation of these impacts.

## Conclusion

In a changing climate, sea level rise and storm surge from potential future storms pose great risks to archaeological sites in coastal regions. Preparing for these potential hazards requires accurate assessments of future risks to heritage sites. An inconsistency between modeled impacts of coastal erosion on archaeological sites largely based on sea level rise and observed effects, largely from individual storm surge events, has thus far hindered our ability to accurately assess the vulnerability of sites to future risks. Our modeling of potential future storm surge events under multiple sea level rise scenarios in the state of Georgia demonstrates that over 4200 archaeological sites along the coast are currently at risk of inundation and erosion from hurricanes, more than ten times as many sites as previously believed to be at risk by 2100 based on analysis of SLR alone. This modeling demonstrates the importance of accounting for storm events rather than gradual SLR alone in cultural heritage management sectors. Our projections are shared with cultural heritage managers in the Georgia Historic Preservation Division in order to facilitate prioritization, documentation, and mitigation efforts. By doing so, we hope to encourage necessary action toward conserving Georgia’s physical cultural heritage. While the case study here focuses on the Georgia Coast, the conditions and approach to modeling can be readily applied to a wide range of coastal environments around the world. Such information, as we demonstrate, is critical for decision makers regarding future conservation, rescue archaeology endeavors, financial planning, and mitigation that is needed before cultural heritage is lost to the next storm.
